# Submicron machining and biomolecule immobilization on porous silicon by electron beam

**DOI:** 10.1186/1556-276X-7-530

**Published:** 2012-09-25

**Authors:** Dario Imbraguglio, Andrea Mario Giovannozzi, Annalisa Nastro, Andrea Mario Rossi

**Affiliations:** 1 Thermodynamics Division, Istituto Nazionale di Ricerca Metrologica, Strada delle Cacce 91, Torino, 10135, Italy

**Keywords:** Porous silicon, Electron beam, Lithography, Micromachining, Biomolecules, 87.85.Va

## Abstract

Three-dimensional submicrometric structures and biomolecular patterns have been fabricated on a porous silicon film by an electron beam-based functionalization method. The immobilized proteins act as a passivation layer against material corrosion in aqueous solutions. The effects' dependence on the main parameters of the process (i.e., the electron beam dose, the biomolecule concentration, and the incubation time) has been demonstrated.

## Background

Electron beam lithography (EBL) is known to be a pattern-designing technique of integrated electrical circuits, with writing resolution even down to tens of nanometers, which allows the fabrication of innovative and advanced devices for nanotechnological applications [1,2]. In EBL procedures, electron-sensitive polymeric resists are usually spun on top of samples' surfaces prior to irradiation in a scanning electron microscope (SEM). Depending on the nature of the resist, the irradiated geometry or its specular negative is used as a mask for subsequent material etching or deposition steps in order to obtain structures with nanometer-scale features. However, the EBL, when directly used to desorb chemical species from a surface, can be also exploited as a local functionalization method [3] to create molecular modified or biopatterns without employing any resist. This has been the case, for instance, of porous silicon (PS) surface-based biosensors [4].

PS is a silicon nanosponge produced by electrochemical etching of crystalline silicon in a hydrofluoric (HF) acid-containing solution. It is one of the most investigated structures in nanomaterials science due to some fascinating properties which can be harnessed in many applied research fields [5]. Morphological parameters of PS matrices, i.e., pore sizes, porosity (voids-to-total volume ratio), and thickness, can be easily controlled just by varying the experimental electrochemical conditions (such as current density, HF concentration, or etching duration), allowing the use of PS for size-selective filtration or separation processes, drug delivery, or sensing applications.

The huge specific surface area (in the order of 200 m^2^/cm^3^) of PS is hydride-terminated in as-etched samples [6,7], and a well-established modification chemistry has been developed in the past years to selectively bind large amounts of different target analytes within the pores [8]. In so far as biological species are concerned, the immobilization of proteins [9], enzymes [10], and antibodies [11] on PS surfaces can be achieved by different techniques. Among the ones which allow submicrometric definitions, the EBL has already proved its capability in designing chemical and biopatterns solely on selected and very small region of PS [3,4], opening the possibility of future development of highly-sensitive nanobiosensors and multiplexed analysis based on this material. From this perspective, PS is a very convenient substrate for EBL processes because of its porous structure. Indeed, due to the lower quantity of Si atoms with respect to an equal volume of bulk Si, PS behaves as a low-density material and exhibits a reduced proximity effect [2], which is the main limitation factor in writing resolution with standard EBL-based methods. Moreover, the absence of the polymeric resist during irradiation further contains the phenomenon because the only cause of overexposure of PS irradiated regions stems from secondary and backscattered electrons just coming from the underlying PS/Si substrate. This means that more defined and small patterned features can be, in principle, obtained by direct, i.e., not resist-mediated, irradiation of PS. Therefore, by combining EBL nanostructuring capabilities with those of PS whose pore dimensions can be varied from the macro- to the microscale (<2 nm), sophisticated optical nanostructures could, in principle, be fabricated, such as three-dimensional (3D) photonic crystals, waveguides, or optical gratings.

As far as the application of the direct EBL method on PS is concerned, some previous works have already demonstrated its capability in material structuring [12,13] as well as the feasibility to fabricate a reliable biosensor [4]. Different kinds of geometries had been patterned on PS surfaces, and the control of feature dimensions is possible by tuning the process parameters. Furthermore, proteins which were bound on PS surfaces by such a resistless EBL technique had been shown to retain their full functionality after the process and can act as bioreceptors for molecular and biomolecular analytes. We report on recent studies and advances on both sides, i.e., the (submicron) machining and the immobilization of biomolecules on PS. The obtained results let us discover a new and intriguing property of the interaction between biomolecular species and a solid-state nanomaterial which, to the best of our knowledge, has never been observed before.

## Methods

PS single-layer films have been anodically etched from highly boron-doped single-crystal Si wafers <100> (resistivity ranging from 0.008 to 0.012 Ω cm) in a 1:2 solution of aqueous 50% HF/ethanol in volume; the electrochemical etching procedure is described in several papers [3-5]. Typically, a double-step etching/stop loop (consisting of 0.2-sec etching at a current density of 400 mA/cm^2^ followed by a 10-sec stop etching time) was repeated, the number of times depending on the desired film thickness, using a Keithley 2400 SourceMeter (Keithley Instruments Inc., Cleveland, OH, USA). PS samples with thicknesses of 6.5 and 9 μm were fabricated, with pore dimensions comprised in the mesoscale regime (approximately 20 to 50 nm) and porosity of about 80%, as confirmed by profilometric and SEM measurements. Anyway, no substantial differences related to the different thickness values of the samples or due to decreasing the current density to 300 mA/cm^2^ were observed in the experimental results.

After dipping in an HF-based solution in order to remove the native silicon oxide layer, PS samples were introduced into the vacuum chamber of a FEI Quanta 3D (FEI Co., Hillsboro, OR, USA) field emission gun (FEG) SEM equipped with a nano pattern generator system (NPGS) 9.0 from J.C. Nabity Lithography Systems (Bozeman, MT, USA). Simple rectangular geometries, formed by line series 0.8-μm wide and 30-μm long (spaced by a distance of 0.7 μm), were written by NPGS on the PS sample surfaces, applying a 20-kV accelerating voltage to the electron beam. The electronic dose (i.e., the number of electrons per area units) range explored in this work has been varied from 40 up to 200 mC/cm^2^.

In order to allow as much consistent comparison between samples as possible, identical patterns were written on specular portions of the same PS chip, which were then divided after irradiation and immediately immersed in buffered solutions. Depending on the test performed, pure 1 X phosphate buffered saline (PBS) or bovine serum albumin (BSA) protein-containing solutions were prepared. PBS tablets and BSA lyophilized powders from Sigma-Aldrich (St. Louis, MO, USA) have been dissolved in purified water provided by a Millipore Elix 3 purification system (Millipore Co., Billerica, MA, USA). The pH of pure PBS solutions has been checked each time by a CyberScan pH/Ion 510 meter (Eutech Instruments, Vernon Hills, IL, USA) to be equal to a nominal value of 7.4. Four different BSA concentrations (5, 1, 0.5, and 0.1 μM) were obtained by multiple dilutions from a 15-μM mother solution of BSA in PBS buffer. The irradiated PS samples were incubated for times ranging from 5 to 120 min, after which they were profusely rinsed first in PBS, then in deionized water and finally dried with nitrogen gas.

Fourier transform infrared (FTIR) reflectance spectra have been acquired using a Nicolet Nexus FTIR spectrophotometer (Thermo Scientific, Logan, UT, USA) coupled with a Nicolet Continuum FTIR microscope equipped with a MCT detector. The spectra were collected both in the irradiated area and just a few micrometers outside by registering a total of 512 interferograms for each spectrum with a resolution of 4 cm^−1^. Finally, the samples were observed with a FEI Inspect F FEG SEM in a tilted (60°) secondary electron configuration.

## Results and discussion

It is well known that high-surface-area nanomaterials such as PS undergo rapid oxidation and even dissolution when exposed to ambient air conditions or immersed in aqueous solutions because of their poor stability [14]. These kinds of processes can be locally further stressed and speeded up by using different techniques, such as laser writing [15,16] or contact atomic force microscopy-based methods [17]. From this point of view, EBL can potentially be used to remove material from a solid substrate only in submicrometer-wide regions due to the high focusing power of the electron beam. Among other different approaches, EBL can be thought of as a good compromise in so far as writing speed and resolution are concerned. As previously mentioned, the interaction between PS and the electron beam has already been studied in some former works [3,4,12,13]. Rocchia et al. [3] first demonstrated that the electron irradiation provokes hydrogen desorption from the native SiH_x_ bonds of PS surfaces, leading to high reactive electron beam-activated PS (EBAPS) regions which easily oxidize in ambient air. Such a strong reactivity is most likely due to the formation of dangling bonds and defects as a consequence of the hydrogen depletion caused by the electron bombardment. Nevertheless, in the reported previous cases, even if the written geometries were clearly distinguishable immediately after irradiation by the electron microscope, optical, profilometric, and SEM investigations did not reveal any substantial structuring of the PS along the *z*-axis (perpendicular to the PS surface). Anyway, very tiny *z*-depth profiles were measured only after removing the electro-oxidized PS areas in acid or alkaline solutions, as the difference step between the top non-exposed PS surface and the bottom crystalline Si one after EBAPS removal. The amplitude of such thicknesses had been studied as a function of different oxidizing conditions (air, water, H_2_O_2_, incubation time) as well as energies and electronic doses provided by the electron beam. The results showed that the stronger the oxidizing procedure is, the thicker is the depth measured by profilometry, and so the final vertical structuring of PS. Besides, monotonic increases in the thickness were observed by augmenting the electronic dose and the accelerating voltage of the electron beam. In the latter case, a 25-kV saturation value was found, beyond which the electrons lose their energy also through the Si bulk underlying the PS substrate, and the effect no longer depends on the electron energy. The maximum depth value (≈180 nm) was obtained with a 12-μm thick sample irradiated at 25 kV at the maximum electronic dose (120 mC/cm^2^) [13]. It has to be pointed out that the reported thickness values were detected only after dipping EBAPS samples in HF or KOH etchants. This is a quite aggressive etching procedure which may cause, in some case, the removal of PS non-exposed areas too due to the low degree of selectivity of such methods. Obviously, as far as micro- or nano-machining of PS is concerned, easiness and precise reproducibility in fabrication of machined structures are two important requirements.

**Figure 1:**
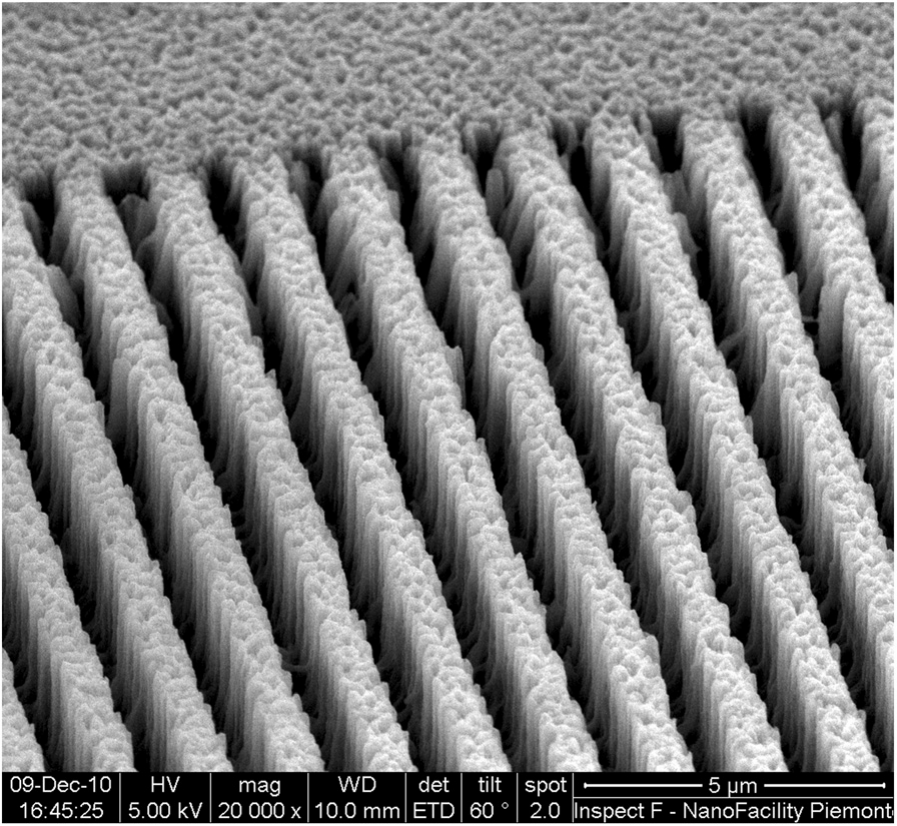
SEM micrograph of an EBAPS sample incubated for 1 h in a pure PBS solution.

Here, our new improvements and results about such a PS EBL-assisted structuring technique are reported. We decided to operate the electron beam with a lower accelerating voltage with respect to the previous cases in order to have a minor penetration depth of electrons through the PS layer. In spite of using high electronic doses even up to 200 mC/cm^2^, we also have not noted any modification on the PS morphological structure just after irradiation. The as-written geometries were barely visible at the optical microscope as well as at the SEM, where the exposed areas were distinguished, even after long storage in air, only by a slight contrast difference in the acquired images. On the other hand, after dipping the EBAPS samples in pure PBS for quite long incubation times, the patterned geometries resulted to be, after the process, as printed onto the PS matrix. Figure 1 displays a SEM tilted image of a sample irradiated with a very low electronic dose (40 mC/cm^2^) and immersed in a pure PBS solution for 1 h. The submicrometric irradiated strips are the void regions in the picture, and an accomplished structuring of the nanomaterial along the *z*-axis is evident. The porous matrix between one strip and another is maintained, revealing that such an erosion effect is doubtlessly caused by the combined action of the buffer only with the EBAPS regions. As we have said, EBL on as-etched PS samples induces hydrogen desorption from the surface, with the consequent formation of very reactive dangling bonds that tend to saturate with other atoms present in the environment, most commonly oxygen. However, PS oxidation in air is not strong enough to produce visible effects on the material, not even by increasing the electronic dose and, therefore, the number of EBAPS sites. In aqueous solutions such as PBS buffer, instead, water molecules can penetrate inside the nanopores, and oxygen atoms link to the EBAPS pore walls. The already weakened and unstable PS nanostructure is most likely further overburdened, and a selective electron beam-driven redox dissolution takes place, which slowly manifests itself in several-minutes-long timescales. In the present case, the corrosion by PBS starts to be noticeable only after about 20 min, while after incubation times up to 1 h, holes with depths of even few micrometers can be easily obtained. By exploiting this process, any 2D geometry can be propagated through the nanostructured ‘PS bulk’ in a way similar to what happens with other emerging bulk Si etching techniques, as for instance metal-assisted-based methods [18]. In the studied submicrometric regime, the 3D fabricated structures are very well defined, and the material removal can be simply controlled by varying the incubation time in an aqueous pH 7.4 or neutral solution.

**Figure 2:**
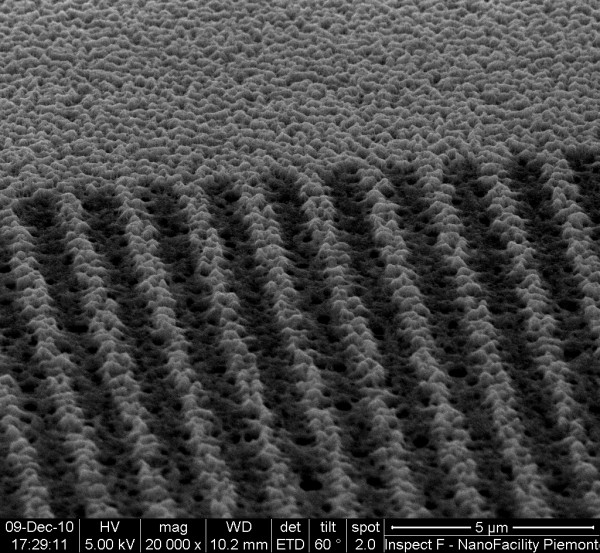
SEM micrograph of an EBAPS sample incubated for 1 h in a 15-μM BSA solution.

More surprisingly, the phenomenon of PS dissolution is in some way limited by the presence of BSA biomolecules in the buffering solutions. As previously mentioned, the enhanced reactivity of EBAPS submicrometric regions can be harnessed to locally bind biomolecules or other chemical species only in the irradiated area. Nevertheless, the anchorage mechanism and dynamics in the solution are still not completely clear, and a deeper study of such immobilization process is currently in progress. As yet, the method seems to be not selective with respect to different biomolecular targets [4]; hence, a huge variety of possibilities can be, in principle, explored to further improve the chemical functionalization skills of PS. We used serum albumin from bovine blood as a test biomolecule since it is relatively small (14 × 3.8 × 3.8 nm^3^, molecular mass ≈ 66 kDa) [19] and able to penetrate inside the pores of our samples. Figure 2 shows a SEM tilted image of PS irradiated in the same conditions with that of the one displayed in Figure 1 but immersed in a 15-μM BSA-containing buffer solution. As clearly shown in Figure 2, the situation is completely different in comparison to the previous case. Here, the irradiated strips are still visible in a SEM image as darker regions across the PS matrix, presumably due to changes in the electrical conductivity of the material, but the overall structure looks almost completely preserved.

**Figure 3:**
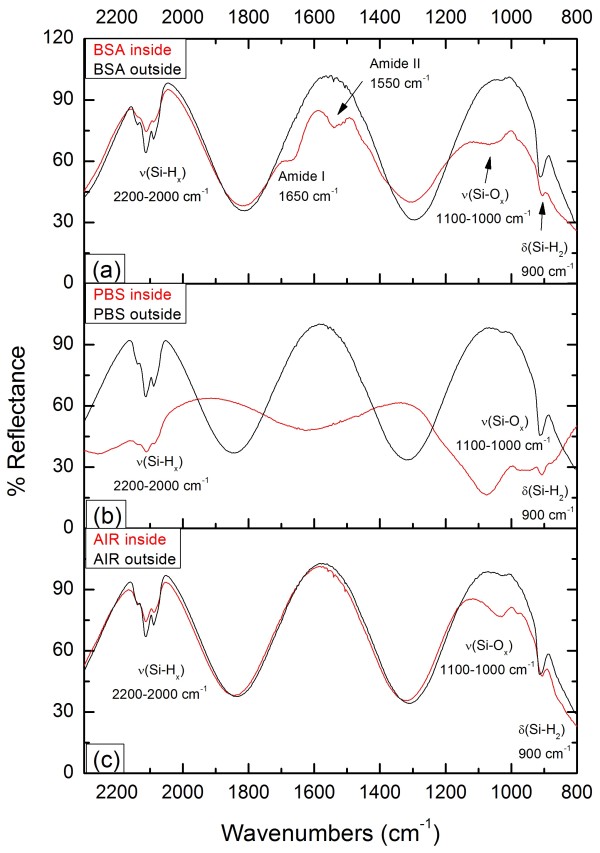
**FTIR spectra of the EBAPS samples.** EBAPS incubated for 1 h in a BSA-PBS solution (**a**), incubated for 1 h in a pure PBS solution (**b**), and stored in air for 1 day (**c**). For each window, the spectrum of the exposed area (red) is compared to the control obtained from the non-exposed one (black).

In order to check the selective immobilization of BSA biomolecules on the PS surface, a systematic FTIR spectroscopic study has been carried out by collecting the reflected infrared radiation from the film inside and outside the EBAPS areas (60 μm × 30 μm). This has been done in the same way for three different typologies of EBAPS samples: (a) EBAPS incubated for 1 h in a BSA-PBS solution, (b) EBAPS incubated for 1 h in a pure PBS solution, and (c) EBAPS left in air atmosphere for 1 day. Figure 3 summarizes the indicative behavior of all the samples by showing the FTIR spectra in the range of 800 to 2,300 cm^−1^. Among the detectable vibrational mode signals, only those related to the adsorption bands of the Si-H_x_ groups (2,000 to 2,200 cm^−1^ and 900 cm^−1^), Si-O_x_ groups (1,000 to 1,100 cm^−1^), and Amide I (1,650 cm^−1^) and Amide II (1,550 cm^−1^) BSA peptide bond [20] have been monitored. As it can be seen from Figure 3, each EBAPS spectrum displays an adsorption signal decrease, with respect to the control obtained outside, of Si-H stretching (2,000 to 2,200 cm^−1^) and scissoring (900 cm^−1^) modes in the electron-irradiated region, which is the hallmark of local hydrogen depletion due to the electron beam activation. It has to be specified that a part of such decrease is also ascribed to a complementary increase of vibrations related to oxidation (1,000 to 1,100 cm^−1^) and, in the case of the samples exposed to the BSA, biomolecular bonding. As expected anyway, the Amide I and Amide II adsorption bands are clearly present only in the spectrum of the irradiated region in the first kind of samples (Figure 3a), meaning that a selective immobilization of proteins has been achieved. We plainly cannot exclude that some small quantities of BSA were also bound to the PS surface between one strip and the other since the FTIR resolution does not allow performing measurements in such a very small area. Nevertheless, the great lateral definition of the strips in Figure 2 suggests that the selective immobilization has been accomplished in the submicron range as well. More important is the different final degree of oxidation displayed by the samples in the spectra of Figure 3. The lowest variation in the Si-O absorption bands between irradiated and non-irradiated regions is the one related to EBAPS stored in air (Figure 3c) for which, as we have previously mentioned, any structural change in the material conformation was observed. This is also confirmed by the maintained periodicity of the Fabri-Pérot interference fringes of the irradiated area by comparing inside and outside regions in the spectra of Figure 3c . In EBAPS samples incubated for 1 h in a BSA-containing buffer solution (Figure 3a), the oxidation is higher but the structure of the PS film is still preserved (Figure 2). On the other hand, the interference pattern uniformity in the FTIR spectra of the EBAPS samples which were left, for the same time, in a BSA-free solution is nearly lost (Figure 3b), and this situation corresponds to the morphological sight of the EBAPS reported in the SEM picture of Figure 1. In this case, the highest degree of oxidation is registered, which let us conclude that the immobilized BSA proteins are able to shield EBAPS surfaces from excessive oxygen binding and thus avoid redox dissolution processes of the PS matrix.

In the present case, the conservation of the Fabri-Pérot interference pattern uniformity has been used as a roundabout optical method to exclude that the preservation of the PS matrix, observed in Figure 2, was due to just a superficial passivation acted by the immobilized proteins. BSA biomolecules are small enough to penetrate inside the nanopores of EBAPS samples as well as water molecules, whose number is, however, several orders of magnitude larger in a few micromolar concentrated BSA buffer solution. It is very unlikely that BSA molecules could directly bind to the activated Si sites through a covalent bond because of, firstly, the highest probability which water molecules have to saturate with EBAPS dangling bonds. Secondly, it had been demonstrated in few cases that the biomolecular functionality of the immobilized target is retained after the process [4], and such a kind of attachment would rather denature the biomolecule of interest. On the other hand, the strong affinity of BSA for Si dioxide is well established [21], and it is therefore plausible that oxygen atoms could act also as a linker for the immobilization of BSA proteins to the EBAPS pore walls. During the incubation time of the EBAPS samples with the BSA solution, two fast reactions probably occurred one after the other: the enhanced oxidation of the irradiated areas and the consequent selective attachment of BSA molecules to these regions. As soon as the reaction proceeds, the constrained space available within the nanopores favors the packaging of the biomolecules, thus creating a sort of scaffold which prevents material corrosion. In this way, a soft matrix as that formed by the BSA proteins is able to protect and sustain a nanostructured solid one.

**Figure 4:**
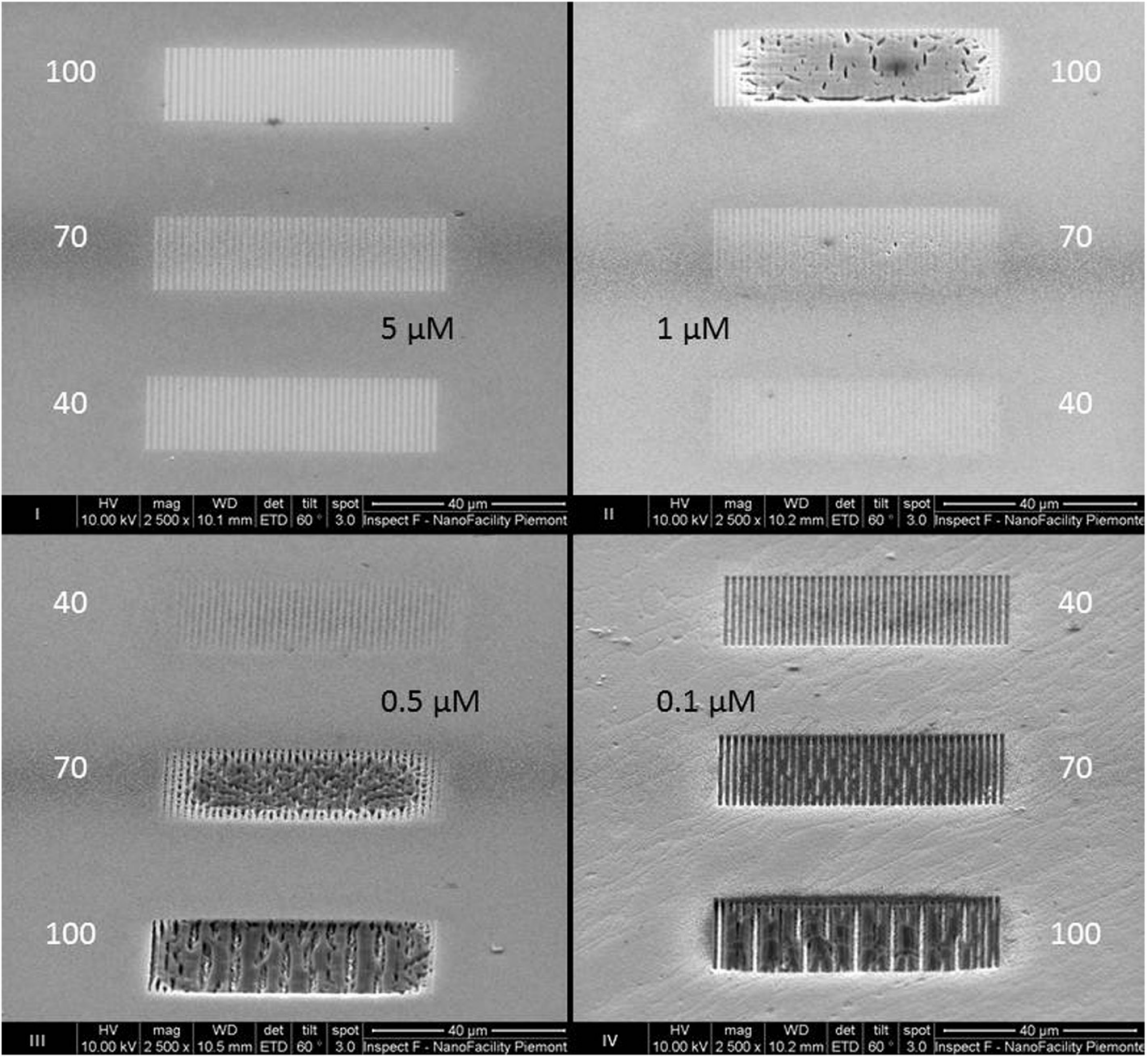
**SEM micrographs of four electron-irradiated regions (I, II, III, and IV) of a PS sample.** Taken after 1-h exposure to distinct BSA solutions. The protein concentrations are indicated (black), as well the increase of the electron beam dose from 40 up to 100 mC/cm^2^ (white).

An interaction mechanism like the one we just described should firstly depend on the initial concentration of biomolecular species in the solution. In addition, considering the dependence of the oxidation on the SEM operating conditions, the PS dissolution process may be further amplified by increasing the electron accelerating voltage or the electronic dose supplied by the electron beam [12,13]. In order to find a final confirmation of that, EBAPS samples were incubated in four protein buffer solutions, each one containing a different concentration of BSA: 5, 1, 0.5, and 0.1 μM. We chose to keep fixed the accelerating voltage at 20 kV and to vary for each sample the electronic dose from 40 up to 70 mC/cm^2^ in order to facilitate quantitative evaluations. Figure 4 groups the SEM tilted images of the four irradiated portions (I, II, III, and IV quadrants in Figure 4) after 1 h of incubation time. Concentration and electronic dose values are indicated in black and white in the figure, respectively. The selective immobilization of BSA and the interferometric pattern features have been even checked in this case by FTIR spectroscopy measurements, which are not reported here. In the reported SEM magnifications, at the highest BSA concentration (5 μM), the biomolecular patterns fabricated by the EBL technique appear as bright strips through the PS matrix (Figure 4, quadrant I). We noted a tiny increase in the strips' breadth by augmenting the electronic dose in such range, which is simply due to a greater proximity effect occurring at high electron beam irradiation conditions. Anyway, the strips are disconnected from one another in every pattern irradiated with a different electronic dose, even at 100 mC/cm^2^, and the submicrometric definition can be achieved by using the lowest one. The situation displayed by the sample incubated in a solution with a lower concentration of BSA (1 μM) is nearly similar (Figure 4, quadrant II), except for some cracks and several-micrometer-square-wide holes which start to compromise the PS structure at the 100-mC/cm^2^ electronic dose. Since these features are not present at 70 mC/cm^2^ as well as at 40 mC/cm^2^, this proves that a higher electronic dose of the electron beam causes greater stress and degradation of the porous material. By further decreasing the BSA concentration down to 0.5 μM, the aspect of the EBAPS samples drastically changes (Figure 4, quadrant III). Here, machined structures begin to form in each electronic dose case. The submicrometric resolution is only accomplished at 40 mC/cm^2^, and PS coalescence phenomena between non-irradiated areas seem to take place at the medium electronic dose, after which the entire exposed and non-exposed regions collapse at the maximum dose. Such a loss of writing resolution is again caused by the increasing proximity effect, which is even more evident by reducing the amount of biomolecules down to the lowest concentration value (0.1 μM; Figure 4, quadrant IV). As it happens in complete absence of biomolecules in the solution (Figure 1), very well-defined rectangular strips that cross the material matrix are produced, in this case, only by using an electronic dose of 40 mC/cm^2^, which seems to be the optimum value to fabricate both submicrometric biomolecular patterns as well as free-standing PS structures. By comparing all the SEM pictures of Figure 4, we therefore demonstrated that the degree of passivation from the electron beam-driven PS dissolution can be controlled by tuning the molar concentration of proteins in solution. Anyway, above a threshold value of 1 μM, the overall EBAPS structure is preserved even at high electronic doses, and stable submicrometric biomolecular patterns can be easily realized. These observations are consistent with some recently published results about the modification of crystalline silicon surfaces by bioactive films of proteins [22], which allow the fabrication of advanced biocompatible hybrid systems. The advantage of our approach lies in the capability to obtain the same passivation effects on an already nanostructured and hence weaker material.

## Conclusions

We found that electron beam irradiation on PS has no noticeable effects on its morphological structure as long as, after EBL writing, samples were left in air or just a few minutes in buffered solutions. On the other hand, if the irradiated PS samples were dipped for incubation times greater than 20 min in pure PBS (or very low BSA concentrated) solutions, the irradiated strips appear, after drying, as well-defined submicrometric vertical structures embedded into the porous matrix, suggesting that a heavy EBL-controlled erosion of the nanomaterial can be accomplished in these conditions. A valuable option to common 3D micro- and nano-machining techniques of PS has thus been proposed.

In addition, submicrometric bio-PS composite patterns have been successfully fabricated by the same technique, and a nanoscale biomolecular passivation effect has also been observed. We are confident to transfer the acquired knowledge to the immobilization of other and more useful nano and biomolecular targets (i.e., conductive biomolecules or functionalized metallic nanoparticles), which could be suitable for applications in different emerging research fields, such as molecular and bioelectronics or surface-enhanced Raman spectroscopy.

## Competing interests

The authors declare that they have no competing interests.

## Authors' contributions

DI carried out the fabrication of the PS samples, the EBL processes, the FTIR characterization, and drafting of the manuscript. AMG participated in the fabrication of the PS samples and FTIR characterization. AN participated in the FTIR characterization. AMR coordinated the study. All authors read and approved the final manuscript.
